# The Menopausal Mind: How Sleep and Immune Function Shape Cognitive Health

**DOI:** 10.1093/geroni/igaf122.1801

**Published:** 2025-12-31

**Authors:** Ashley Curtis, Amy Costa, Natasa Billeci, Melanie Stearns, Fareeha Hussaini, Amy Brown, Christina McCrae

**Affiliations:** University of South Florida, Tampa, Florida, United States; University of South Florida, Tampa, Florida, United States; University of South Florida, Tampa, Florida, United States; University of South Florida, Tampa, Florida, United States; University of South Florida, Tampa, Florida, United States; University of South Florida, Tampa, Florida, United States; University of South Florida, Tampa, Florida, United States

## Abstract

Sleep health and poor immune functioning are linked to worse cognition. There are known sex differences in both functions (2x higher insomnia prevalence and increased low grade inflammation in aging women). However, whether interactive associations impact cognition across the menopausal transition remains understudied. This study examined interactive associations between insomnia symptoms and immune status on cognition in mid-life women. Middle-aged women with insomnia and cognitive complaints (N = 51, aged 42-64 years) completed the Insomnia Severity Index, Immune Status Questionnaire, Cognitive Failures Questionnaire (CFQ) and Everyday Memory Questionnaire at baseline and 3-month follow-up. Multiple regression analyses examined interactive associations between proportional changes in insomnia severity, immune status and cognition, and evaluated whether these were further moderated by menopause status (STRAW +10 pre/peri/post staging), controlling for age. Menopause status significantly moderated interactive insomnia severity/immune functioning associations with CFQ-distractibility (R-squared=.08, p=.04), with a trend for CFQ-Total (R-squared=.07, p=.07). Specifically, worsening of insomnia severity was associated with increased distractibility only in women who experienced greatest decline in immune functioning and were either peri-menopausal (B=.43±.18, p=.02) or post-menopausal (B=.53 ± =.22, p=.02). Similarly, worsening insomnia severity was associated with increased global cognitive complaints in peri-menopausal women with moderate (B=.33±.16, p=.047) or greatest (B=.50±.20, p=.02) decline in immune functioning, and in peri-menopausal women with greatest immune functioning decline (B=.57±.25, p=.02). Sleep and immune responses may synergistically interact with sex-hormone disruption during menopausal transition and impact neurocognitive health. Findings point to sex-specific sleep and associated mechanistic targets for early monitoring and intervention to mitigate risk of cognitive decline and/or neurodegeneration.

